# Aerith: Visualization and Annotation of Isotopic Enrichment Patterns of Peptides and Metabolites with Stable Isotope Labeling from Proteomics and Metabolomics

**DOI:** 10.1021/acs.analchem.5c03207

**Published:** 2025-10-28

**Authors:** Yi Xiong, Ryan S. Mueller, Shichao Feng, Xuan Guo, Chongle Pan

**Affiliations:** † School of Biological Sciences, 6187 University of Oklahoma, Norman, Oklahoma 73019-0390, United States; ‡ Department of Microbiology, 549630 Oregon State University, Corvallis, Oregon 97331-1311, United States; § Department of Computer Science and Engineering, 53417 University of North Texas, Denton, Texas 76203-5017, United States; ∥ School of Computer Science, University of Oklahoma, Norman, Oklahoma 73019-0390, United States; ⊥ Stephenson School of Biomedical Engineering, University of Oklahoma, Norman, Oklahoma 73019-0390, United States

## Abstract

Stable isotope probing (SIP) traces the metabolism of biological cells using isotopically heavy substrates (e.g., ^13^C, ^15^N, or ^2^H). Confident identification of the metabolic products of isotopic labeling remains a challenge due to the difficulties in simulating, visualizing and annotating the isotopic patterns of partially labeled peptides and metabolites found in mass spectrometry (MS) data. Here, we present Aerith, an R package designed to visualize data of simulated and observed isotopic envelopes of peptides and metabolites with user-defined formula and atom % enrichment levels. Aerith models the isotopic distributions of the fragment ion series of a peptide by sequentially convoluting isotopic envelopes of monomeric units using a convolution algorithm. Aerith simulates fine isotopic structures of a compound using Monte Carlo simulation via the multinomial distribution, and calculates the isotopic envelopes of metabolites with known chemical formulas using an FFT-based algorithm. These algorithms provide accurate simulation of the isotopic envelopes of SIP-labeled peptides and metabolites with high computational efficiency. Aerith evaluates peptide-spectrum matches through multiple robust and commonly used scoring functions to compare experimental and theoretical spectra. These algorithms were implemented in C++ and accessed in R via Rcpp to ensure real-time interactivity and significantly improve computational efficiency compared to native R code. We present case studies to demonstrate Aerith’s utility in resolving isotopic fine structures and envelopes for glucose, penicillin, and microbial peptides containing natural and enriched isotopes. By providing visualization of isotopically labeled peptides and metabolites, Aerith enables precise annotation of their mass spectra and manual validation of their identifications in proteomic and metabolomic SIP studies.

## Graphical abstract

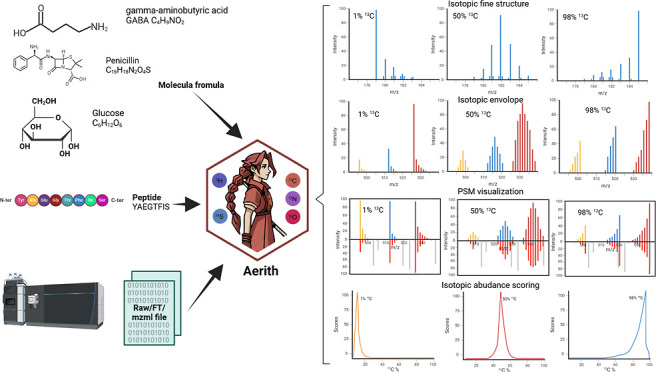

## Introduction

Stable isotopes can be used as tracers for metabolic fluxes in biological and biogeochemical systems, providing their quantitation in complex biological contexts.^1^ Modern mass spectrometry, including Fourier-transform mass spectrometry (FTMS) and time-of-flight mass spectrometry (TOF-MS), allows precise measurement of isotope distributions and fine structures at high resolution.^2^ To support these applications, specialized R packages have been developed for stable isotope fingerprinting (SIF) and isotope ratio mass spectrometry (IRMS). For example, simmr^3^ employs a Bayesian framework to model stable isotope mixing in food-web studies for ecological tracer analysis. The isoverse software suite integrates isoreader^4^ for data parsing and isoorbi^5^ for isotope calculations from Orbitrap data. The assignR^6^ facilitates geographic provenance mapping using isotopic signatures. IsoSpec2 ^7^ provides rapid simulation of isotopic fine structures under natural abundance conditions, but it does not support analysis of compounds with partially isotope incorporation from stable isotope probing (SIP) experiments.

SIP has provided novel insights into the diversity and functions of microbial communities within various biogeochemical cycles.^8–10^ The Miso R package^11^ supports metabolomic analyses through multi-isotope labeling workflows, but it requires the isotopic envelope of the known 100% isotopically enriched molecular tracer. In contrast, proteomic SIP tracks stable label incorporation into proteins—such as enzymes or structural proteins in microbial cells—via LC–MS/MS and enrichment-resolved database searches. This allows taxonomic and functional characterization of labeled substrate assimilation by microbial communities based on peptide-spectrum matches (PSM) from metaproteomic measurements. Proteomic SIP has been developed to investigate the metabolic functions of microbiomes^12,13^ in diverse environments, including single-strain incubations,^14^ hot spring microbial mats,^15^ coastal seawater,^16^ the mouse gut,^17^ biogas digesters,^18^ groundwater^19^ and anaerobic bioreactors.^20^ Several proteomic SIP tools—such as Sipros,^21,22^ MetaProSIP,^23^ Calisp^10^ have been developed to analyze partially labeled peptide mixtures; however, they only report estimated labeling percentages without further interpretation. PDV^24^ enables visualization of proteomic search results in pepXML^25^ and mzid formats, but it does not allow user-defined amino acid sequences or molecular formulas for isotopically labeled peptides and compounds.

To address these limitations, we developed Aerith, a computational tool for simulation of theoretical isotopic peaks, fine structures, and spectral envelopes of user-defined chemical formulas or peptide sequences in metabolomics or proteomics at both the MS and MS/MS levels. The software allows user-defined isotopic abundances of SIP-labeled elements across all enrichment levels. Aerith enables visualization and manual validation of unlabeled and SIP-labeled peptide-spectrum matches (PSMs) from proteomic SIP data.

## Feature and Implementation

### Input Data Format

Aerith accepts spectral data files in multiple formats, including Raxport-processed FT2, mzML, and MGF formats, as well as pepXML and PIN files (Percolator outputs), TSV files from the Sipros search engine^21,22^ for peptide amino acid sequences or PSM input. The functions imported from mzR^26^ enables direct parsing of mzML and MGF files. All imported spectra are available for downstream analysis. Figure 1 summarizes key metadata of input spectra processed by Aerith. Users may also input peptide sequences or chemical formulas to simulate isotopic envelopes with adjustable isotopic abundances (e.g., ^13^C, ^2^H, ^18^O, ^15^N, ^34^S) for theoretical spectral analysis.

**Figure 1:**
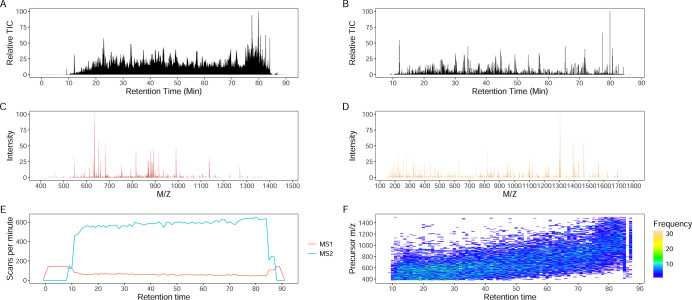
Visualization of an LC–MS/MS run in Aerith. An unlabeled *E. coli* sample was analyzed in data-dependent acquisition (DDA) mode. (A) Total ion chromatogram (TIC) of MS1 scans. (B) TIC of MS2 scans. (C) MS1 scan at the retention time of 74.04 min. (D) Data-dependent MS2 scan on *m*/*z* 950.9609 with a 5 Da isolation window of the MS1 scan shown in C. (E) Scan acquisition rates for MS1 (red) and MS2 (blue) levels. (F) Precursor selection frequencies across *m*/*z* and retention time.

### Theoretical Spectra Generation for SIP-Labeled Metabolites

Theoretical spectra with isotopic fine structures are generated using a Monte Carlo algorithm which repeatedly simulates multinomial isotope distributions and, thus, is able to capture low-abundance fine-structure variants albeit at high computational cost (Attachment S1; Figure 2A–D). To obtain better computational efficiency, Aerith also incorporates an FFT-based algorithm to produce isotopic envelopes. This algorithm retains only the main peaks separated by integer neutron differences, transforms each elemental distribution via FFT, multiplies them pointwise according to stoichiometry, and applies an inverse FFT to obtain the aggregate envelope^27^ (Attachment S2; Figure 2E,F). The users may select either the Monte Carlo algorithm for isotopic fine structures or the FFT-based algorithm for rapid envelope computation. Both algorithms accept user-defined chemical formulas and specified abundances (ranging from 0% to 100%) of stable isotope-labeled elements (e.g., ^13^C, ^15^N) as input. The resulting theoretical spectra can be exported as tabulated data, including *m*/*z*, charge state, and intensity values.

**Figure 2:**
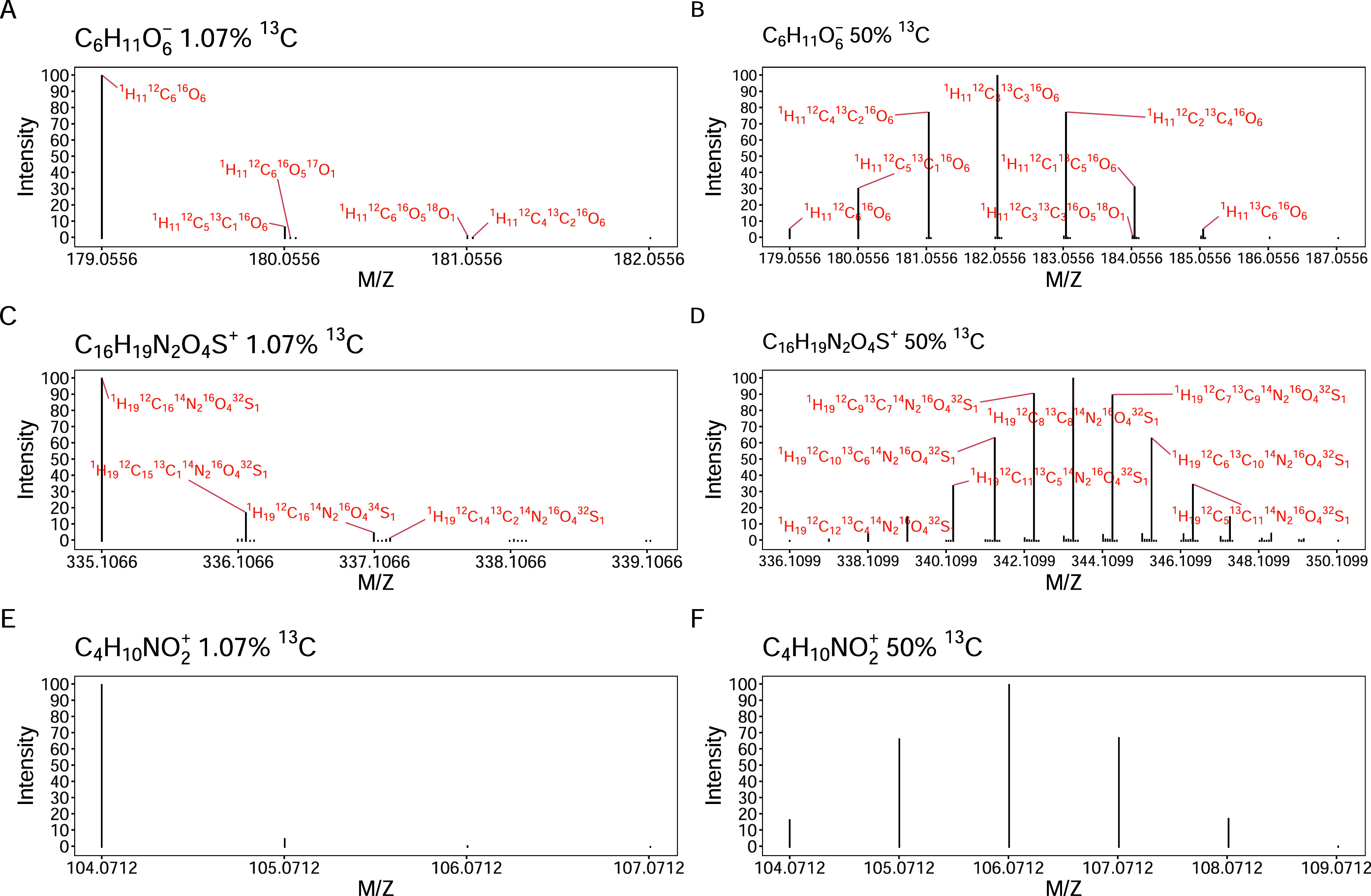
Simulated isotopic fine structures and envelopes of SIP-labeled metabolites. The isotopic fine structures of negative-ion mode glucose ([M – H]^−^) are simulated at 1.07% (natural abundance) (A) and 50% ^13^C enrichment (B), which can be compared with their measured spectra from MZcloud (Thermo Fisher) (Figure S3). Adjacent isotopic peaks with minor *m*/*z* differences (<0.03 Da) were computationally offset while preserving true positional relationships for visual clarity. High-abundance isotopologues are annotated in red with their isotopic compositions. The isotopic fine structures of positive-ion mode penicillin ([M + H]^+^) at 1.07% (natural abundance) (C) and 50% ^13^C enrichment (D), validated against reference spectra from MZcloud (Figure S4). The isotopic envelopes (ignoring fine structure) of positive-ion mode γ-aminobutyric acid ([M + H]^+^) at 1.07% (natural abundance) (E) and 50% ^13^C enrichment (F) were generated using the FFT algorithm in comparison with experimental metabolomics data from natural abundance and SIP-labeled samples.^28^

### Generation of Theoretical Spectra for Tandem Mass Spectrometry of SIP-Labeled Peptides in Bottom-Up Proteomics

Aerith simulates the isotopic envelopes of peptide precursors and b/y fragment ions using a convolution algorithm that combines the mass distributions of isotopologues from individual amino acid residues (Attachment S5). Aerith also implements three PSM scoring functions: MVH (multivariate hyper-geometric distribution),^29^ Xcorr (cross-correlation),^30^ WDP (Weighted dot product)^21,22^ (Attachments S6–S8). All these algorithms were implemented in C++ for computational efficiency and integrated into the R environment via the Rcpp interface to simplify usage and improve accessibility. Given the amino acid sequence of a peptide and its isotopic abundances of SIP-labeled elements (e.g., ^13^C, ^15^N) provided by the user, theoretical spectra without isotopic fine structures are generated using a convolution-based algorithm^31^ combined with binomial distribution estimation (Attachment S9). Figure 3 illustrates isotopic envelopes simulated by Aerith for a representative peptide (HYAHVDCPGHADYVK) under natural (1.07% ^13^C) and enriched (50% ^13^C) conditions.

**Figure 3:**
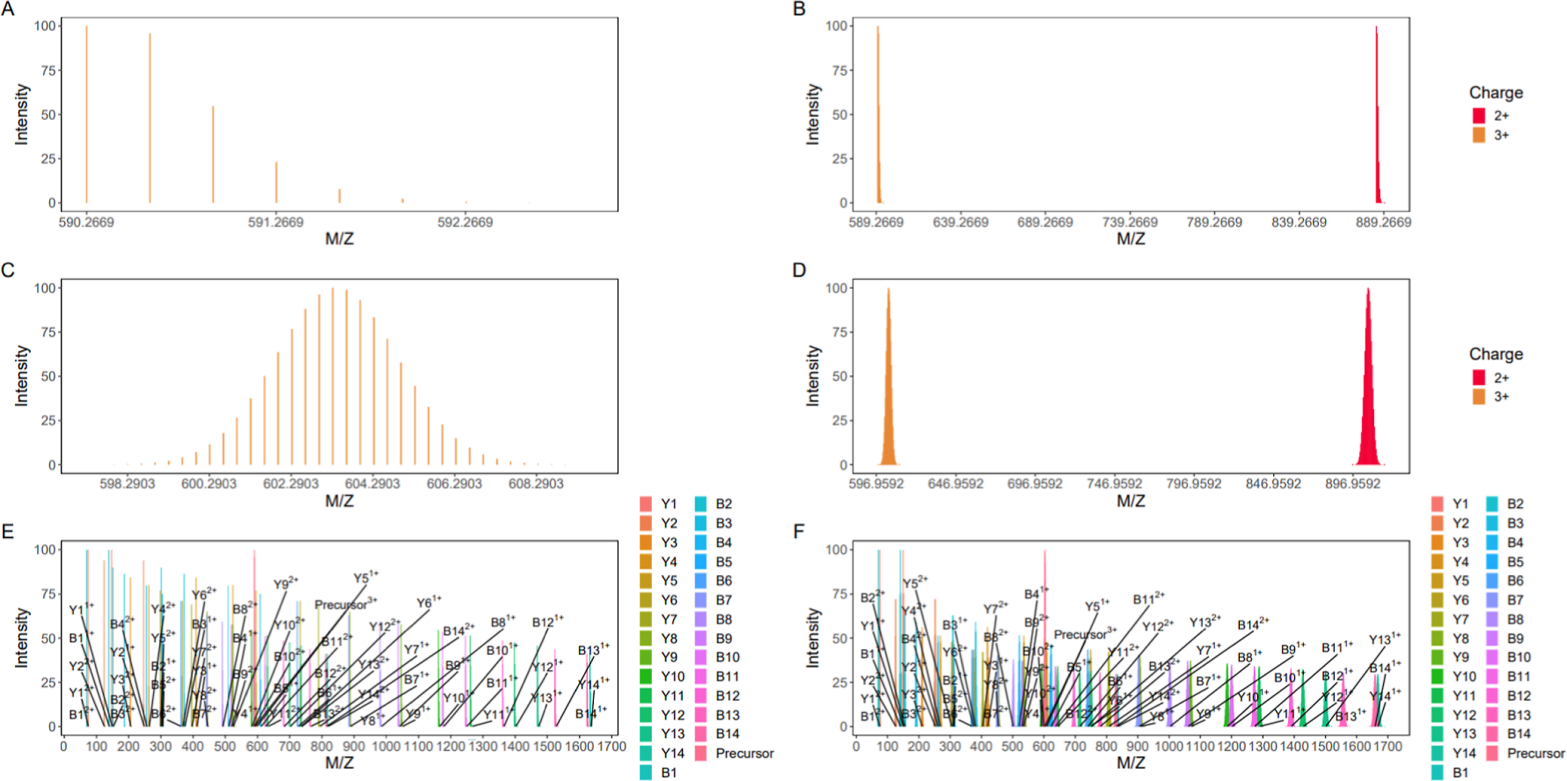
Simulated isotopic envelopes of SIP-labeled peptide precursors and fragment ions. (A) Precursor ion (charge state +3) at 1.07% ^13^C (natural abundance). (B) Precursor ions (charge states +2 [red] and +3 [yellow]) at 1.07% ^13^C. (C) Precursor ion (charge state +3) at 50% ^13^C enrichment. (D) Precursor ions (charge states +2 [red] and +3 [yellow]) at 50% ^13^C. (E) B/Y fragment ions (charge states +1/+2) at 1.07% ^13^C. (F) B/Y fragment ions (charge states +1/+2) at 50% ^13^C.

### Visualization of SIP-Labeled and Unlabeled PSM

Aerith employs ggplot2 to provide publication-quality visualization of PSMs. Figure 4 compares PSMs of the peptide HYAHVDCPGHADYVK with natural abundance (1.07% ^13^C) and 50% ^13^C-abundance, as identified by Sipros. It visualizes *m*/*z* shifts from ^13^C enrichment in both precursor and fragment ions. The visualization requires two spectral inputs: observed spectra (optionally preprocessed via a denoising function to reduce stochastic noise interference) and theoretical spectra. The output of the visualization function includes a composite S4 object of PSM containing matched ion metadata, spectral data, and customizable ggplot2 layers in R environment.

**Figure 4:**
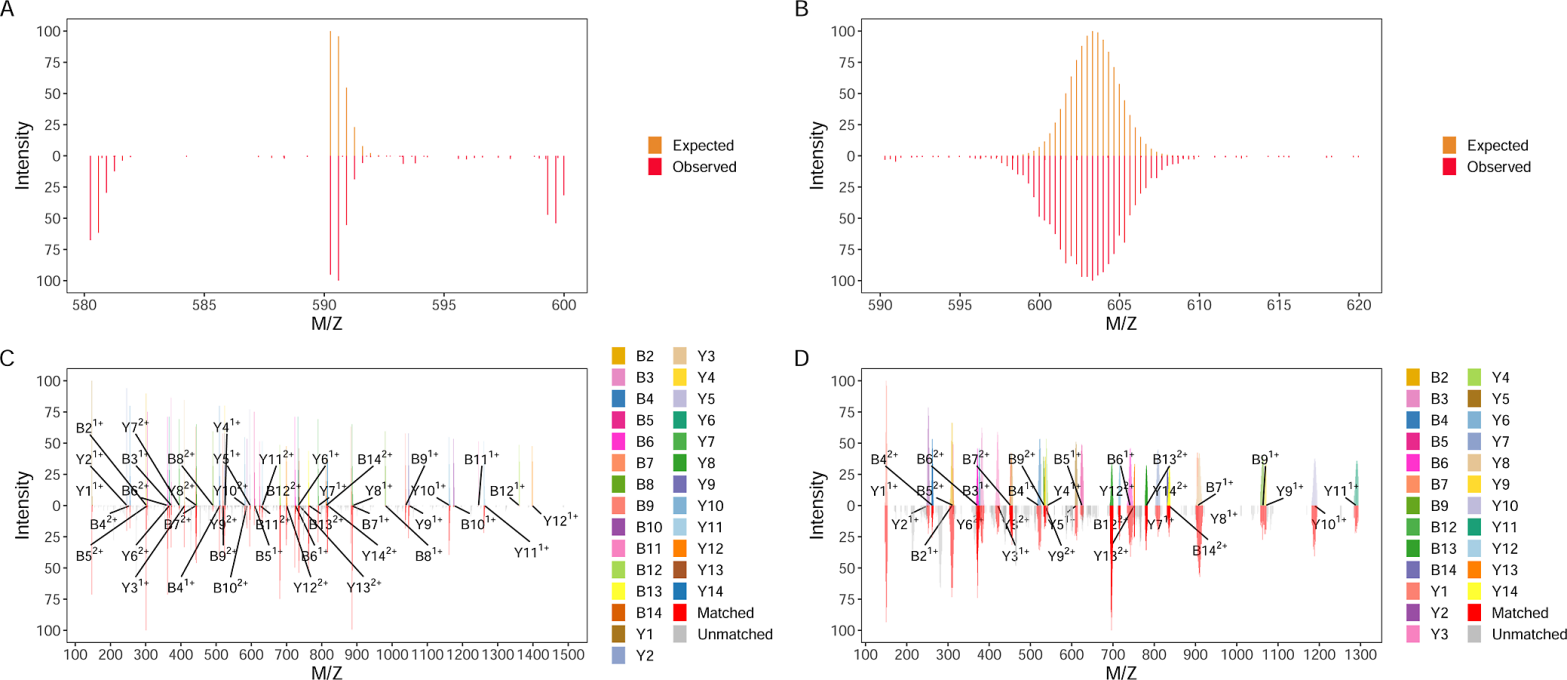
Visualization of PSMs with observed spectra in the lower panel and theoretical isotopic peaks in the upper panel. The highest-matching isotopic peaks within the envelope are highlighted. B/Y fragment ions are annotated based on user preferences. (A) Precursor PSM at 1.07% ^13^C (natural abundance), with theoretical envelope (yellow) and observed peaks (red). (B) Precursor PSM at 50% ^13^C that demonstrates the mass shift from isotopic enrichment. (C) B/Y ion PSM at 1.07% ^13^C; unmatched peaks are annotated in gray. (D) B/Y ion PSM at 50% ^13^C.

### Performance Evaluation of PSM Scoring Functions

The PSM can be quantitatively assessed using three scoring functions, including MVH, Xcorr, WDP, and spectral entropy (Attachment S10).^32^ Figure 5 demonstrates the results of these three scoring functions and spectral entropy for the peptide HYAHVDCPGHADYVK under natural (1.07% ^13^C) and enriched (50% ^13^C) isotopic conditions. Among these, the WDP scoring function, originally developed for the Sipros algorithm demonstrates the narrowest peak width in score distributions across 0–100% ^13^C abundance levels, indicating superior resolution in distinguishing true matches. Users may also implement custom scoring algorithms via the package interface.

**Figure 5:**
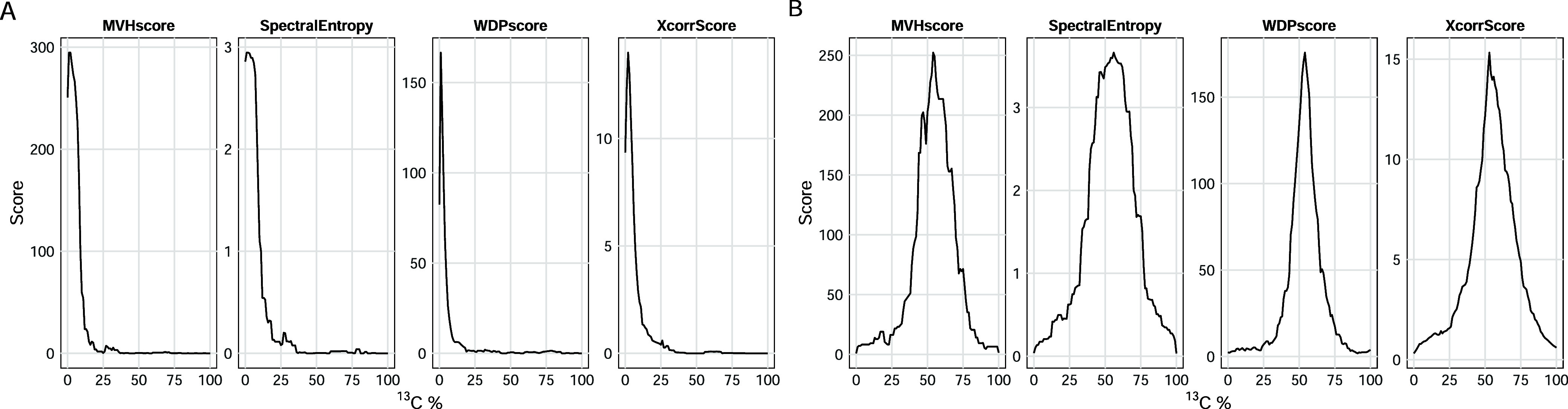
Comparison of scoring functions for estimation of ^13^C enrichment levels from PSMs. (A) Scores for the peptide at 1.07% ^13^C (natural abundance). (B) Scores for the peptide at 50% ^13^C enrichment.

## Results and Discussion

Aerith presented in this study establishes a robust computational framework for simulating isotopic patterns with a modular C++/R architecture. The package provides three distinct methods for theoretical isotopic envelope generation, each suited to specific applications. The FFT method achieves the highest computational speed with the lowest algorithmic complexity (Supporting Information S2); however, it does not account for isotopic fine structure, which can be resolved experimentally via high-resolution mass spectrometry (HRMS) or simulated using the Monte Carlo method (Supporting Information S1). For small molecules or metabolites with known chemical formulas or structures, the FFT method is optimal. In contrast, the Monte Carlo method is preferable when isotopic fine structure must be considered, such as in the analysis of clumped isotopes.^33^ For polymers like peptides or proteins, the convolution-based method efficiently computes isotopic envelopes for fragment ions without recomputing each from scratch (Supporting Information S5).

Aerith significantly enhances proteomic stable isotope probing (SIP) workflows by enabling systematic validation of database search outputs. For scoring peptide-spectrum matches (PSMs), the weighted dot product (WDP) function outperforms other methods for SIP-labeled PSMs (Figure 5) due to its comprehensive evaluation of all isotopic peaks in fragment ions (Supporting Information S6). In contrast, when analyzing low-resolution mass spectra (e.g., ion trap or time-of-flight (TOF) MS^2^ measurements of peptide fragments), incomplete isotopic envelopes favor the XCorr and MVH scoring functions. MS^2^ scans obtained in data-independent acquisition (DIA) mode can also result in incomplete isotopic envelopes. These two scoring functions are particularly suited to this situation. This preference can be explained by the fact that both methods prioritize the most abundant isotopic peak and account for noise-induced random matches, which are also common in TOF or Astral^34^ scans (Supporting Information S7, S8). Prior studies have demonstrated spectral entropy as a critical feature for scoring small-molecule spectral identifications,^32^ suggesting that implementing entropy-based scoring (Supporting Information S10) could advance metabolite identification in SIP-metabolomics. Furthermore, Aerith streamlines user-defined and automatic mass spectral annotation, supporting validation of critical findings in proteomic SIP studies.

The resources associated with this work are publicly available. The Aerith R package for spectral analysis is accessible at https://github.com/thepanlab/Aerith. The raw file conversion tool Raxport can be downloaded from https://github.com/xyz1396/Raxport.net. For SIP proteomics searches, the Sipros program is hosted on GitHub (https://github.com/thepanlab/Sipros4) and Bioconda (https://anaconda.org/bioconda/sipros). Case study raw files are deposited in the PRIDE Archive under accession PXD041414 (https://www.ebi.ac.uk/pride/), and the corresponding analysis scripts are embedded within the Aerith package vignettes.

## Supplementary Material



## Notes

This Supporting Information file provides additional methods, algorithms, reference spectra, and scoring details that complement the main manuscript (PDF)
