# Supplementary material for: Aerith: Visualization and Annotation of Isotopic Enrichment Patterns of Peptides and Metabolites with Stable Isotope Labeling from Proteomics and Metabolomics
Source: Anal Chem. 2025 Oct 28;97(45):24916–21. doi: 10.1021/acs.analchem.5c03207 (PMC12631728; doi:10.1021/acs.analchem.5c03207)
Supplement: Supplementary file 1 [file ac5c03207_si_001.pdf]

Aerith: visualization and annotation of isotopic enrichment patterns of peptides and metabolites with stable isotope labeling from proteomics and metabolomics

Yi Xiong[1], Ryan S. Mueller[2], Shichao Feng[3], Xuan Guo[3], and Chongle Pan[1,4,5,*]

[1]School of Biological Sciences, University of Oklahoma, Norman, OK
[2]Department of Microbiology, Oregon State University, Corvallis, OR
[3]Department of Computer Science and Engineering, University of North Texas, Denton, TX
[4]School of Computer Science, University of Oklahoma, Norman, OK
[5]Stephenson School of Biomedical Engineering, University of Oklahoma, Norman, OK

*Address correspondence to: cpan@ou.edu

This Supporting Information file provides additional methods, algorithms, reference spectra, and scoring details that complement the main manuscript.

Table of Contents

S1 Isotopic fine structure calculation for chemical formulas 2

S2 Isotopic envelopes calculation using FFT 3

Figure S3. Reference spectra from MZcloud of negative-ion mode glucose ($[M\text{-}H]^-$) at 1.07% (natural abundance) ^{13}C enrichment. 6

Figure S4. Reference spectra from MZcloud of positive-ion mode penicillin ($[M+H]^+$) at 1.07% (natural abundance) ^{13}C enrichment. 6

S5 Isotopic envelopes calculation by convolution for peptide sequences.................... 7

S6 WDP score calculation for peptide-spectrum match (PSM) 9

S7 MVH Score Calculation for PSM 12

S8 Xcorr Score Calculation for PSM.. 15

S9 Peptide precursor mass estimation using binomial distributions 17

S10 Spectral Entropy Calculation for PSM 19

S1 Isotopic fine structure calculation for chemical formulas

Description

This code calculates isotopic envelopes of molecules by simulating natural or modified isotopic distributions using Monte Carlo sampling. The approach involves parsing a chemical formula into element counts, randomly sampling isotopes for each element based on natural or specified abundances, running thousands of simulations to build a statistical distribution of isotopic compositions, calculating the mass and relative abundance of each unique isotopic composition, and visualizing the results as a mass spectrum.

Key Equations

The code parses formulas like $C_6H_{12}O_6$ into element counts using regular expressions.

For each element E with n atoms and isotopes i_1, i_2, ..., i_k with abundances p_1, p_2, ..., p_k:

$$[n_1, n_2, \ldots, n_k] \sim Multinomial(n, [p_1, p_2, \ldots, p_k]) \quad (1)$$

Where $n_1 + n_2 + \ldots + n_k = n$ (total atoms of element E).

For a molecule with specific isotopic composition:

$$Mass = \sum(n_i \times m(i_i)) \quad (2)$$

Where n_i is the count of isotope i and $m(i_i)$ is its mass.

$$Abundance(composition) = Count(composition) \,/\, Total\_Simulations \quad (3)$$

Pseudocode

```
Input:
  formula_str    – string, e.g. "C6H12O6"
  N              – integer, number of Monte Carlo trials (default 10000)
  params         – named overrides, e.g. { C13 = 0.5, N15 = 0.2 }

Output:
  isotope_numbers – data frame of simulated isotope counts + masses + abundances

1. Save original abundances
    original_abundances ← copy(shared_env$isotopic_abundances)

2. Apply SIP overrides to abundances
    for each (iso_name, new_abund) in params:
      if iso_name ∈ names(shared_env$isotopic_abundances):
        # set target isotope to user value
        shared_env$isotopic_abundances[iso_name] ← new_abund
```

```
            # redistribute the remaining fraction among its sibling isotopes
            element_symbol ← strip_digits(iso_name)
            siblings ← supported_isotopes[element_symbol] \ {iso_name}
            remaining ← 1 – new_abund
            # scale siblings to sum to remaining
            total_sib_abund ← sum(original_abundances[siblings])
            for each sib in siblings:
                shared_env$isotopic_abundances[sib] ←
                    original_abundances[sib] / total_sib_abund * remaining
        else:
            warning("Unsupported isotope override:", iso_name)

3. Run Monte Carlo simulation
    # this calls cal_isotope_numbers() which parses the formula,
    # draws random isotope counts per element based on shared_env,
    # then summarizes into counts, masses, abundances
    isotope_numbers ← cal_isotope_numbers(formula_str, N)

4. Restore original abundances
    shared_env$isotopic_abundances ← original_abundances

5. Return isotope_numbers
```

The strength of this approach is its ability to simulate realistic isotopic distributions that match experimental mass spectra, especially for complex molecules with elements having multiple naturally occurring isotopes.

S2 Isotopic envelopes calculation using FFT

Description

This algorithm implements a Fast Fourier Transform (FFT) approach to calculate isotopic distributions of molecules. The method approximates the isotopic pattern of a molecule based on its chemical formula by breaking down the formula into individual elements and their quantities, using the natural abundance of each isotope to calculate probability distributions, Applying FFT to efficiently convolve these distributions, and converting back to the mass domain using inverse FFT. The key advantage of this approach is computational efficiency when handling large molecules with many atoms, as direct convolution would be prohibitively expensive.

Key Equations

For a molecule with multiple elements, the isotopic distribution is the convolution of the isotopic distributions of each element, raised to the power of the number of atoms of that element.

Let's define:

$P_X(i)$ = probability of element X having i additional neutrons

n_X = number of atoms of element X in the molecule

$P_{mol}(k)$ = probability that the molecule has k additional neutrons

The basic convolution equation is:

$$P_{mol}(k) = \underbrace{P_C * P_C * \ldots * P_C}_{n_C \text{ times}} * \underbrace{P_H * P_H * \ldots * P_H}_{n_H \text{ times}} * \ldots \quad (4)$$

Using the Fourier transform property that convolution in one domain equals multiplication in the Fourier domain:

$$\mathcal{F}[P_{mol}] = \mathcal{F}[P_C]^{n_C} \cdot \mathcal{F}[P_H]^{n_H} \cdot \ldots \quad (5)$$

Then using the inverse Fourier transform to get back the distribution:

$$P_{mol} = \mathcal{F}^{-1}[\mathcal{F}[P_C]^{n_C} \cdot \mathcal{F}[P_H]^{n_H} \cdot \ldots] \quad (6)$$

Pseudocode

```
Input:
    formula_str        : string, e.g. "C6H6O"
    N_width            : integer, FFT vector length (must cover full isotope envelope)
    min_abundance      : float, filter threshold for low-abundance peaks
    …                  : named overrides for individual isotope abundances

Output:
    mass_bins          : array of peak m/z (or mass) values
    probabilities      : array of normalized abundances

1. Parse the formula into element counts
    element_array ← parse_chemical_formula(formula_str)
      # e.g. {"C":6, "H":6, "O":1}

2. (Optional) override single-isotope abundances via … parameters
    for each param in …:
        if param matches an isotope name:
            adjust shared_env$isotopic_abundances[param] and rescale its element's other
                isotopes

3. Compute two key mass constants
    monoisotopic_mass ← ∑[element, count] ( count ×
        mass_of_most_abundant_isotope(element) )
      # exact integer masses (e.g. C12 = 12.0000, H1 = 1.0078, O16 = 15.9949)
    average_neutron_mass ← calculate_average_neutron_mass(element_array)
      # weighted mean Δmass between heavier and lighter isotopes per neutron
```

```
4. Build the FFT-based abundance envelope
    # a) initialize frequency-domain accumulator
    abundance_fft ← 1
    # b) for each element with multiple isotopes
    for each (element, count) in element_array:
        if count > 0 and element has >1 isotope:
            # get its natural-abundance vector, zero-pad to length N_width
            iso_vec ← shared_env$isotopic_abundances[ all isotopes of element ]
            padded_vec ← pad_with_zeros(iso_vec, N_width)
            # FFT → raise to the power 'count' → multiply into running product
            abundance_fft ← abundance_fft × [ FFT(padded_vec) ]^count
    # c) invert, take real part, normalize
    raw ← IFFT(abundance_fft)
    probs ← Re(raw) / sum(Re(raw)) # probabilities P(k) for k = 0…N_width−1 extra neutrons

5. Translate neutron-count bins into masses
    # each index k corresponds to k extra neutrons beyond the monoisotopic peak
    mass_bins ← array of length = length(probs)
    for k from 0 to length(probs)-1:
        mass_bins[k] ← monoisotopic_mass + k × average_neutron_mass

6. Filter out tiny peaks and return
    keep ← probs > min_abundance
    return mass_bins[keep], probs[keep]
```

The code approximates the mass difference between isotopes as one neutron mass, which simplifies calculations but ignores fine structure like mass defects. The most elegant aspect is using FFT to convert convolution operations (which are computationally expensive) into multiplications in the frequency domain. The approach scales well for large molecules where direct convolution would be impractical. The implementation allows custom isotope abundances to be specified (like changing C^{13} abundance), enabling applications in stable isotope probing experiments. The method provides a fast approximation of isotopic distributions suitable for many practical applications in mass spectrometry and biochemistry.

Figure S3. Reference spectra from MZcloud (https://beta.mzcloud.org/, Thermo Fisher) of negative-ion mode glucose ([M-H]$^-$) at 1.07% (natural abundance) ^{13}C enrichment.

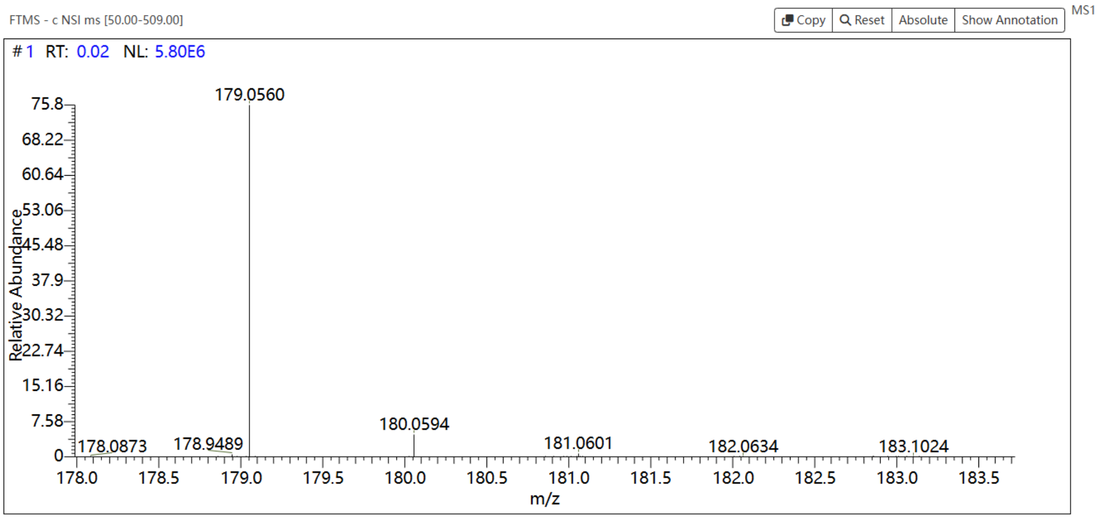

Figure S4. Reference spectra from MZcloud of positive-ion mode penicillin ([M+H]$^+$) at 1.07% (natural abundance) ^{13}C enrichment.

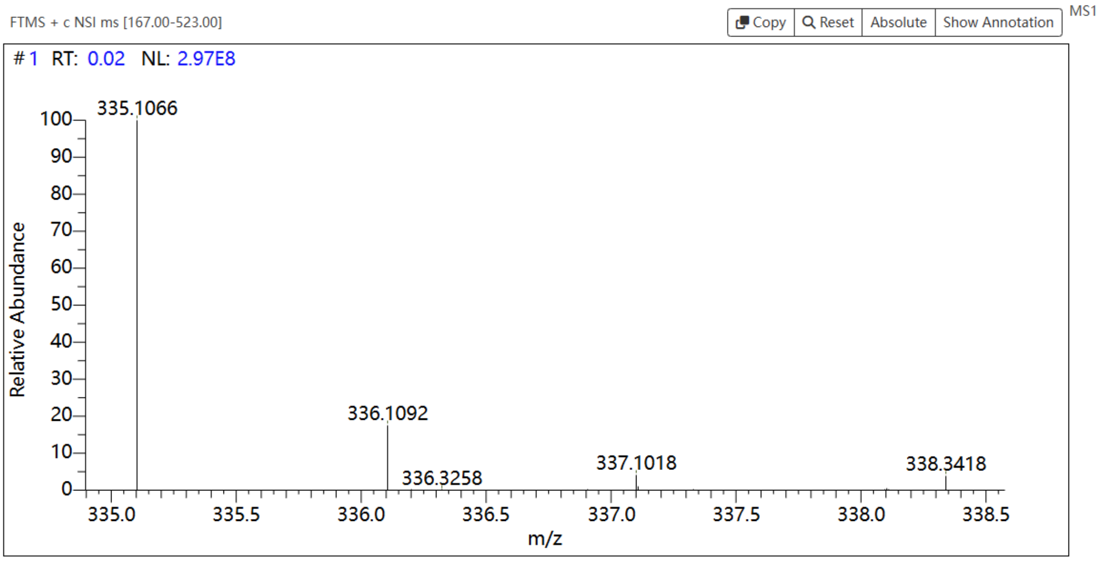

S5 Isotopic envelopes calculation by convolution for peptide sequences

Description

The code implements algorithms for calculating the isotopic distribution (envelope) of peptides by convolving the isotopic distributions of individual amino acid residues and chemical elements. The primary functions involved in this calculation are \`computeIsotopicDistribution\`, \`sum\`, and \`multiply\`.

Key Equations

For two isotopic distributions D_0 andD_1, each represented by a set of masses and corresponding probabilities, the convolution operation is defined as:

$$D_{\text{sum}} = D_0 \otimes D_1 \tag{7}$$

Where D_{sum} is the resulting combined distribution.

For a given peak index k in the resulting distribution, where $k \in \{0,1,2,\dots,n_0+n_1-2\}$:

$$p_{\text{sum}}(k) = \sum_{i=\max(0,k-n_1+1)}^{\min(n_0-1,k)} p_0(i)\cdot p_1(k-i) \tag{8}$$

$$m_{\text{sum}}(k) = \frac{\sum_{i=\max(0,k-n_1+1)}^{\min(n_0-1,k)} p_0(i)\cdot p_1(k-i)\cdot\left(m_0(i)+m_1(k-i)\right)}{\sum_{i=\max(0,k-n_1+1)}^{\min(n_0-1,k)} p_0(i)\cdot p_1(k-i)} \tag{9}$$

Where n_0 is number of peaks in distribution D_0, n_1 is number of peaks in distribution D_1 , $p_0(i)$ is probability of peak i in distribution D_0, $p_1(k-i)$ is probability of peak $k-i$ in distribution D_1, $m_0(i)$is mass of peak i in distribution D_0, $m_1(k-i)$is mass of peak $k-i$ in distribution D_0, i is index in the first distribution, range: $[\max(0,k-n_1+1),\min(n_0-1,k)]$, $k-i$ is corresponding index in the second distribution.

For calculating the isotopic distribution of multiple copies of the same atom/residue:

$$D_{\text{product}} = D_0^{\otimes c} \tag{10}$$

Where c is the count of atoms of that type.

This is implemented by iterative application of the convolution operation:

$$D_{\text{product}} = \underbrace{D_0 \otimes D_0 \otimes \dots \otimes D_0}_{c\ \text{times}} \tag{11}$$

For a peptide with sequence $S = [r_1, r_2, \dots, r_L]$, the complete isotopic distribution is calculated as:

$$D_{\text{peptide}} = D_{\text{N-term}} \otimes D_{\text{C-term}} \otimes D_{r_1} \otimes D_{r_2} \otimes \dots \otimes D_{r_L} \tag{12}$$

For a B-ion of length j $(where\ 1 \leq j < n)$:

$$D_{B_j} = D_{r_1} \otimes D_{r_2} \otimes \ldots \otimes D_{r_j} \quad (13)$$

For a Y-ion of length j $(where\ 1 \leq j < n)$:

$$D_{Y_j} = D_{Nterm} \otimes D_{Cterm} \otimes D_{r_n} \otimes D_{r_{n-1}} \otimes \ldots \otimes D_{r_{n-j+1}} \quad (14)$$

Where D_{B_j} is the isotopic distribution of the B-ion of length j, $D_{Nterm}$is the isotopic distribution of the N-terminus, D_{Cterm} is the isotopic distribution of the C-terminus, D_{r_i} is the isotopic distribution of the ith residue, $\otimes$ represents the convolution operation. This systematic application of convolutions allows the calculation of isotopic envelopes for molecules of arbitrary complexity.

Pseudocode

```
function computeIsotopicDistribution(atomicComposition, resultDistribution):
    # Input:
    #     atomicComposition: vector of counts for each atom type (C,H,O,…)
    #     vAtomIsotopicDistribution: array of atomic isotope distributions
    # Output:
    #     resultDistribution: final convolved isotopic distribution from atom counts
    sumDistribution ← multiply(vAtomIsotopicDistribution[0], atomicComposition[0])
    # first atom
    for i from 1 to atomicComposition.length–1:
    # remaining atom types
        currentDistribution ← multiply(vAtomIsotopicDistribution[i], atomicComposition[i])
        sumDistribution ← sum(sumDistribution, currentDistribution)
    resultDistribution ← sumDistribution    # return result

function sum(distribution0, distribution1):
    # Input:
    #     distribution0, distribution1: two isotope distributions to convolve
    # Output:
    #     sumDistribution: their convolution (masses averaged, probabilities summed)
    newSize ← distribution0.size + distribution1.size – 1
    sumDistribution ← new IsotopeDistribution(newSize)
    for k from 0 to newSize–1:
        sumWeight ← 0; weightedMassSum ← 0
        start ← max(0, k – distribution1.size + 1)
        end ← min(distribution0.size – 1, k)
        for i from start to end:
```

```
                j ← k – i
                w ← distribution0.prob[i] * distribution1.prob[j]
                sumWeight ← sumWeight + w
                weightedMassSum ← weightedMassSum + w * (distribution0.mass[i] +
distribution1.mass[j])
            if sumWeight > 0:
                currentMass ← weightedMassSum / sumWeight        # weighted average mass
            else:
                currentMass ← average(distribution0.mass, distribution1.mass)    # fallback
            currentProb ← sumWeight
            sumDistribution.mass[k] ← currentMass
            sumDistribution.prob[k] ← currentProb
        return sumDistribution

function multiply(distribution, count):
    # Input:
    #     distribution: single-atom isotope distribution
    #     count: integer copies (can be negative)
    # Output:
    #     productDistribution: self-convolution of ‘distribution’ count times
    if count == 1:
        return distribution
    productDistribution ← deltaDistribution()    # peak at mass 0, prob 1
    else:
        for t from 1 to count:
            productDistribution ← sum(productDistribution, distribution)
    return productDistribution
```

S6 WDP score calculation for peptide-spectrum match (PSM)

Description

The WDP (Weighted Dot Product) score is a method for evaluating the quality of a peptide-spectrum match (PSM) by analyzing the isotopic envelopes of matched fragment ions. This scoring function combines multiple aspects of peptide fragmentation, including mass accuracy, isotopic pattern similarity, charge state correctness, and presence of complementary ion pairs. The WDP score is calculated by iterating through all matched ion isotopic envelopes and computing a score contribution for each matched fragment ion. These contributions are summed to get the final score.

Key Equations

For each matched ion isotopic envelope k:

$$\mathrm{WDP}_k = s_k \cdot c_k \cdot h_k \cdot g_k \tag{15}$$

Where: s_k is the isotopic envelope score, c_k is the charge state penalty, h_k is the mass accuracy score, g_k is the complementary fragment bonus.

The total WDP score is:

$$\mathrm{WDP} = \sum_k \mathrm{WDP}_k \tag{16}$$

The mass accuracy score h_k reflects how well the observed mass matches the expected mass:

$$h_k = 2 \cdot \left(1 - 0.5 \cdot \left(1 + \mathrm{erf}\left(\frac{\mathrm{avgMassError}}{\mathrm{massAccuracyThreshold}/2 \cdot \sqrt{2}}\right)\right)\right) \tag{17}$$

Where $\mathrm{avgMassError} = \frac{\sum_{i=1}^{n} |m/z_{\mathrm{observed},i} - m/z_{\mathrm{expected},i}|}{n}$, $\mathrm{massAccuracyThreshold} = \mathrm{tolerancePPM} \times 10^{-6} \times m/z_{\mathrm{expected}}$.

Isotopic envelope score (s_k) measures how well the observed isotopic pattern matches the expected pattern:

$$s_k = 1 + \sum_{i=1}^{n-1} e_i \tag{18}$$

Where e_i is calculated for each isotope peak (except the monoisotopic peak):

$$e_i = 0.5 - 0.5 \cdot \mathrm{erf}\left(\frac{\left|I_{\mathrm{expected},i}^{\mathrm{rel}} - I_{\mathrm{observed},i}^{\mathrm{rel}}\right|}{\sqrt{\left(I_{\mathrm{expected},i}^{\mathrm{rel}}\right)^2 + \left(I_{\mathrm{observed},i}^{\mathrm{rel}}\right)^2}}\right) \tag{19}$$

Where $I_{\mathrm{expected},i}^{\mathrm{rel}} = \frac{I_{\mathrm{expected},i}}{I_{\mathrm{expected},0}}$, $I_{\mathrm{observed},i}^{\mathrm{rel}} = \frac{I_{\mathrm{observed},i}}{I_{\mathrm{observed},0}}$, I is the intensity of isotopic peak.

Charge state penalty (c_k):

$$\mathrm{c_k} = \begin{cases} 1 & \text{if expectedcharge} = \text{observedcharge} \\ 0.5 & \text{otherwise} \end{cases} \tag{20}$$

Complementary fragment bonus (g_k):

$$g_k = \begin{cases} 2 & if\ complementary\ fragment\ exists \\ 1 & otherwise \end{cases} \tag{21}$$

Pseudocode

```
Input:
    matchedIonIsotopicEnvelopes: map<(ionType, residuePos) → list<Match>>
        # Match has observedMZ, expectedMZ, observedIntensity, expectedIntensity,
observedCharge, expectedCharge
    tolerancePPM: float
    peptideLength: int
```

```
Output:
    WDPscore: float

Initialize total score
    WDPscore ← 0.0

For each (ionType, residuePos), matches in matchedIonIsotopicEnvelopes:
    # skip ions with no observed peaks
    if matches is empty:
        continue

    # Compute average mass error over all isotopic peaks
    sumMassError ← 0.0
    for each match in matches:
        sumMassError ← sumMassError + |match.observedMZ - match.expectedMZ|
    avgMassError ← sumMassError / matches.size()

    # Convert tolerance from PPM to absolute ΔM
    #     ΔM = tolerancePPM·1e-6·m
    massAccuracyThreshold ← tolerancePPM * 1e-6 * matches[0].expectedMZ

    # Compute mass-error weighting h_k via error function
    h_k ← 2.0 * (1.0
                  - 0.5 * (1.0 + erf( avgMassError / (massAccuracyThreshold/2.0)
                    / sqrt(2.0) )))

    # Compute isotope-envelope shape score s_k
    s_k ← 1.0
    for i from 1 to matches.size()-1:
        expRel ← matches[i].expectedIntensity / matches[0].expectedIntensity
        obsRel ← matches[i].observedIntensity / matches[0].observedIntensity
        # e_i penalizes deviation between expected and observed relative intensities
        e_i ← 0.5 - 0.5 * erf( |expRel - obsRel|
                                / sqrt(expRel^2 + obsRel^2) )
        s_k ← s_k + e_i

    # Charge-state consistency factor
    if matches[0].expectedCharge == matches[0].observedCharge:
        c_k ← 1.0
    else:
        c_k ← 0.5

    # Complementary fragment bonus g_k
```

```
        compType ← (ionType == 'B') ? 'Y' : 'B'
        compPos ← peptideLength - residuePos
        if (compType, compPos) in matchedIonIsotopicEnvelopes:
            g_k ← 2.0    # complementary fragment observed
        else:
            g_k ← 1.0

        # Accumulate weighted score
        WDPscore ← WDPscore + s_k * c_k * h_k * g_k

# Return final score
    return WDPscore
```

S7 MVH Score Calculation for PSM

Description

The MVH (multivariate hyper-geometric) score is a statistical measure used to evaluate the quality of peptide-spectrum matches (PSMs) in mass spectrometry-based proteomics. The MVH score calculates the statistical significance of a peptide-spectrum match using a hypergeometric probability distribution. It measures how likely it is to observe the match between theoretical and experimental peaks by random chance. The score is expressed as the negative logarithm of this probability, meaning higher scores indicate more significant matches.

Key Equations

The hypergeometric probability distribution is used to calculate the p-value of a match:

$$P(X \geq n\_match) = \sum_{j=n\_match}^{n\_theo} P(X = j) \tag{22}$$

Where each probability term is calculated as:

$$P(X = j) = \frac{\binom{n\_theo}{j}\binom{N-n\_theo}{n\_obs-j}}{\binom{N}{n\_obs}} \tag{23}$$

N is the number of all possible peak positions after discretization, *n_theo* is the number of theoretical peaks, *n_obs* is the number of observed peaks, *n_match* is the number of matched peaks.

For numerical stability, the code computes the logarithm of the binomial coefficients using the gamma function:

$$\log\binom{n}{k} = \log\Gamma(n+1) - \log\Gamma(k+1) - \log\Gamma(n-k+1) \tag{24}$$

The final MVH score is calculated as:

$$\text{MVH} = -\log_{10}\big(P(X \geq n\_match)\big) \tag{25}$$

Pseudocode

```
Input:
   realScan→mz           # observed m/z values
   expectedMZs           # theoretical m/z values
   residuePositions      # residue index for each expected m/z
   ionKinds              # ion type (B, Y, …) for each expected m/z
   matchedIndices        # index of matched observed peak or –1
   tolerancePPM          # ppm tolerance
Output:
   MVHscore              # –log10 of tail probability

# Find the observed m/z range
    maxMz ← max(realScan→mz)
    minMz ← min(realScan→mz)

# Compute width of one tolerance bin (in Da)
    toleranceWidth ← ((maxMz + minMz)/2) * tolerancePPM/1e6 * 2

# Total number of non-overlapping bins across the range
    N ← (maxMz – minMz) / toleranceWidth

# Initialize counters
    n_theo ← 0               # total theoretical peaks considered
    n_match ← 0              # total matched peaks
    n_obs    ← len(realScan→mz)
    topN     ← 3             # peaks per isotopic envelope
    ix       ← 0
    nISO     ← 0             # count per residue
    lastResiduePosition ← residuePositions[0]

# Sweep through all expected peaks
    while ix < len(expectedMZs):
        if residuePositions[ix] ≠ lastResiduePosition:
            lastResiduePosition ← residuePositions[ix]
            nISO ← 0             # reset for new residue

        if minMz ≤ expectedMZs[ix] ≤ maxMz:
            if ionKinds[ix] ∈ {B, Y}:
                n_theo ← n_theo + topN
```

```
        # count up to topN matched peaks per residue
        if matchedIndices[ix] ≠ –1 AND nISO < topN:
          n_match ← n_match + 1
          nISO    ← nISO + 1

      ix ← ix + 1

# Sum the hypergeometric tail from j = n_match to n_theo
   pValue ← 0.0
   for j in [n_match … n_theo]:
      pValue ← pValue + hypergeomProbability(n_theo, n_obs, j, N)

# Avoid log(0)
   if pValue < 1e–300:
      pValue ← 1e–300

# Return
MVHscore ← –log10(pValue)

Function hypergeomProbability(n_theo, n_obs, j, N):
Input: n_theo, n_obs, j, N
Output: one-term hypergeometric probability

# invalid draws
   if j > n_theo OR (n_obs–j) > (N–n_theo):
      return 0.0

# use log-space for stability
   logTerm ← logBinom(n_theo, j)
            + logBinom(N–n_theo, n_obs–j)
            – logBinom(N, n_obs)

return exp(logTerm)

Function logBinom(n, k):
Input: n, k
Output: log (C(n,k))

if k < 0 OR k > n:
      return –∞            # invalid binomial

# lgamma(x) = log((x–1)!)
```

```
return lgamma(n+1) – lgamma(k+1) – lgamma(n–k+1)
```

S8 Xcorr Score Calculation for PSM

Description

The XCorr (cross-correlation) score is a widely utilized metric in proteomics for assessing the similarity between an experimental spectrum and a theoretical spectrum derived from a peptide sequence. To prepare both observed and theoretical spectra, normalize the intensities and convert the m/z values. Next, bin both spectra into discrete intervals based on a specified tolerance window. Calculate the correlation at zero shift (direct correlation). Then, compute the average correlation at multiple non-zero shifts to estimate random matches. Finally, subtract the average shifted correlation from the zero-shift correlation to obtain the final XCorr score.

Key Equations

The correlation at shift s is defined as:

$$C(s) = \sum_{j \in B_T} B_T[j] \times B_O[j+s] \tag{26}$$

where $B_T[j]$ is the intensity in sparse bin j of the theoretical spectrum, and $B_O[j+s]$ is the intensity in sparse bin $j+s$ of the observed spectrum.

The XCorr score is then calculated as:

$$\text{XCorr} = C(0) - \frac{1}{2W}\sum_{s=-W, s\neq 0}^{W} C(s) \tag{27}$$

Where W is Shift window (75 bins in both directions), $C(0)$ is the correlation at zero shift, $\frac{1}{2W}\sum_{s=-W, s\neq 0}^{W} C(s)$ is the average correlation over all non-zero shifts within the window.

Pseudocode

```
# Input:
#     observedSpectrum: list of {mz, intensity, charge}
#     peptideSequence: string
#     tolerancePPM: float
# Output:
#     xcorr: float

function calculateXCorr(observedSpectrum, peptideSequence, tolerancePPM):
    # Preprocess observed peaks: convert to single-charge m/z and strip proton mass
    obsMz ← empty list
    obsIntensity ← empty list
    for each peak in observedSpectrum:
```

```
        if peak.charge == 0:
                correctedMz ← peak.mz − protonMass
        else:
                # multiply by charge then subtract total proton mass
                correctedMz ← peak.mz * peak.charge − peak.charge * protonMass
        obsMz.append(correctedMz)
        obsIntensity.append(peak.intensity)

# Normalize observed intensities to [0,1]
maxObs ← max(obsIntensity)
for i from 0 to length(obsIntensity)−1:
        obsIntensity[i] ← obsIntensity[i] / maxObs

# Generate theoretical spectrum for the peptide
theoMz, theoIntensity ← empty lists
generateTheoreticalSpectrum(peptideSequence, theoMz, theoIntensity)
    # e.g. fragment ions, normalize to unit max

# Define binning parameters and extend range for shifts
minMz ← min(obsMz)
maxMz ← max(obsMz)
binWidth ← tolerancePPM/1e6 * (minMz + maxMz)/2
shiftWindow ← 75
minMz ← max(minMz − shiftWindow*binWidth, 0)
maxMz ← maxMz + shiftWindow*binWidth

# Bin both spectra into sparse maps: {binIndex → max intensity}
theoBins ← binSpectrum(theoMz, theoIntensity, binWidth, minMz, maxMz)
obsBins ← binSpectrum(obsMz, obsIntensity, binWidth, minMz, maxMz)

# Compute zero-shift correlation
cc0 ← correlationAtShift(theoBins, obsBins, shift=0)

# Compute average correlation over all non-zero shifts
sumShifted ← 0
count ← 0
for shift from −shiftWindow to +shiftWindow:
        if shift != 0:
                sumShifted ← sumShifted + correlationAtShift(theoBins, obsBins, shift)
                count ← count + 1
avgShift ← sumShifted / count

# XCorr is the difference, floored at zero
```

```
    xcorr ← cc0 − avgShift
    if xcorr < 0:
        xcorr ← 0
    return xcorr

# Builds a sparse bin map by assigning each peak to floor((m−minMz)/binWidth)
# and keeping only the maximum intensity per bin.
function binSpectrum(mzList, intensityList, binWidth, minMz, maxMz):
    bins ← empty map<int,double>
    for i from 0 to length(mzList)−1:
        m ← mzList[i]
        inten ← intensityList[i]
        if m < minMz or m > maxMz:
            continue
        binIndex ← floor((m − minMz) / binWidth)
        # retain the strongest peak in each bin
        if binIndex not in bins or inten > bins[binIndex]:
            bins[binIndex] ← inten
    return bins

# For a given shift, multiplies paired bin intensities and sums.
function correlationAtShift(theoBins, obsBins, shift):
    sumCorr ← 0
    for each (binIndex, theoInt) in theoBins:
        shiftedIndex ← binIndex + shift
        if shiftedIndex in obsBins:
            sumCorr ← sumCorr + theoInt * obsBins[shiftedIndex]
    return sumCorr
```

The code optimizes the calculation by using sparse bins (only storing non-zero intensity bins) to avoid wasting memory and computation on empty m/z regions.

S9 Peptide precursor mass estimation using binomial distributions

Description

This method was proposed to calculate the mass with the highest probability within the isotopic envelope of a peptide precursor. This method accounts for the natural abundance of isotopes and potential SIP. It employs a binomial distribution approximation to estimate the contribution of all isotopes to the peptide mass.

Key Equations

In a binomial distribution with parameters n (number of trials) and p (probability of success), the maximum probability occurs at the mode, which is:

$$k_{mode} = \lfloor (n+1)p \rfloor \quad (28)$$

Where $\lfloor x \rfloor$ represents the floor function (greatest integer not exceeding x). For cases where $(n+1)p$ is an integer, both $\lfloor (n+1)p \rfloor$ and $\lfloor (n+1)p \rfloor - 1$ have equal probability. In the code implementation, the function approximates precursor mass by using:

$$M_{precursor}(P) = M_{base}(P) + \text{round}\left(n_k(P) \cdot p_k^{SIP}\right) \cdot \Delta m_k \tag{29}$$

Where $M_{base}(P)$ is the base mass using only the lightest isotopes, $round$ rounds to the nearest integer, $n_k(P)$is the number of atoms of element k in peptide P, p_k^1 is the natural abundance probability of the heavy isotope, p_k^{SIP} is the enrichment level of the SIP element, Δm_k is the neutron mass difference.

Pseudocode

```
# Input:
#     baseMass: float,    monoisotopic base mass of the peptide
#     atomCount: int, number of atoms of the SIP element in the peptide
#     neutronMass: float,    mass shift per neutron
# Globals:
#     SIPelement: string, one of "C", "H", "O", "N", "S"
#     pepAtomCounts: map, e.g. pepAtomCounts["C"] gives total C count
#     C13Abundance, H2Abundance, O18Abundance, N15Abundance, S34Abundance: float
# Output:
#     estimated precursor mass (float)

function estimatePrecursorMassbyNP(baseMass, atomCount, neutronMass):
    # Read current SIPelement
    # Compute number of heavy-isotope substitutions:
        if SIPelement == "C":
            # only C-13 from labeled carbon
            expected ← round(atomCount * C13Abundance)

        else if SIPelement == "H":
            # H-2 labeling plus natural C-13 background
            expected ← round( atomCount * H2Abundance
              + pepAtomCounts["C"] * C13Abundance
            )

        else if SIPelement == "O":
            # O-18 has 2 extra neutrons each + C-13 background
            expected ← round( 2 * atomCount * O18Abundance
              + pepAtomCounts["C"] * C13Abundance
            )
```

```
        else if SIPelement == "N":
            # N-15 labeling + C-13 background
            expected ← round(atomCount * N15Abundance
               + pepAtomCounts["C"] * C13Abundance
            )

        else if SIPelement == "S":
            # S-34 has 2 extra neutrons each + C-13 background
            expected ← round(2 * atomCount * S34Abundance
               + pepAtomCounts["C"] * C13Abundance
            )

        else:
            # no labeling
            expected ← 0

    # Apply mass shift
        precursorMass ← baseMass + expected * neutronMass

    Return precursorMass
```

By using this binomial distribution approximation, the code provides a computationally efficient way to estimate the contribution of heavier isotopes to the peptide mass without calculating the full isotope distribution for each peptide.

S10 Spectral Entropy Calculation for PSM

Description

This function calculates the entropy score for matched spectral peaks in a PSM. Entropy measures spectral complexity or information content. The function calculates the total intensity of all peaks in the real scan. For each matched peak, it computes the probability by dividing the peak's intensity by the total intensity. The entropy is then calculated as the negative sum of the probability times the log of the probability for all matched peaks. The entropy score helps evaluate how information is distributed across matched peaks, with higher entropy indicating a more complex distribution of peak intensities among matched fragments.

Key Equations

$$H = -\sum_{i \in \text{matched}} p_i \log(p_i)$$

Where H is the spectral entropy score, $p_i = \frac{I_i}{\sum_j I_j}$ is the probability of peak i, I_i is the intensity of peak i.

Pseudocode

```
# Input:
#     real_intensities: array of float,    realScan->intensity
#     matched_indices: array of int, matchedIndices
# Output:
#     entropy_score: float, matchedSpectraEntropyScore

# Initialize accumulators
    totalIntensity ← 0.0
    entropy ← 0.0

# Sum all observed intensities to get normalization constant
    for i from 0 to length(real_intensities) - 1 do
        totalIntensity ← totalIntensity + real_intensities[i]
    end for

# For each matched peak, compute its probability and add its contribution to entropy
    for i from 0 to length(matched_indices) - 1 do
        if matched_indices[i] ≠ -1 then
            # Retrieve intensity of the matched peak
            intensity ← real_intensities[ matched_indices[i] ]
            # Compute probability p = intensity / totalIntensity
            prob ← intensity / totalIntensity
            # Accumulate Shannon entropy term: –p · log(p)
            entropy ← entropy + (–prob * log(prob))
        end if
    end for

# Store or return the final entropy score
    entropy_score ← entropy
```
